# Principles and Applications of Two-Dimensional Semiconductor Material Devices for Reconfigurable Electronics

**DOI:** 10.3390/nano15030201

**Published:** 2025-01-27

**Authors:** Jiong Pan, Yike Zhang, Jiaju Yin, Pengwen Guo, Yi Yang, Tian-Ling Ren

**Affiliations:** 1 School of Integrated Circuits, Tsinghua University, Beijing 100084, China; 2 Beijing National Research Center for Information Science and Technology (BNRist), Tsinghua University, Beijing 100084, China; 3 Weiyang College, Tsinghua University, Beijing 100084, China

**Keywords:** two-dimensional materials, two-dimensional semiconductors, reconfigurable electronics, reconfigurable logic, artificial intelligence

## Abstract

With the advances in edge computing and artificial intelligence, the demands of multifunctional electronics with large area efficiency are increased. As the scaling down of the conventional transistor is restricted by physical limits, reconfigurable electronics are developed to promote the functional integration of integrated circuits. Reconfigurable electronics refer to electronics with switchable functionalities, including reconfigurable logic operation functionalities and reconfigurable responses to electrical or optical signals. Reconfigurable electronics integrate data-processing capabilities with reduced size. Two-dimensional (2D) semiconductor materials exhibit excellent modulation capabilities through electrical and optical signals, and structural designs of 2D material devices achieve versatile and switchable functionalities. 2D semiconductors have great potential to develop advanced reconfigurable electronics. Recent years witnessed the rapid development of 2D material devices for reconfigurable electronics. This work focuses on the working principles of 2D material devices used for reconfigurable electronics, discusses applications of 2D-material-based reconfigurable electronics in logic operation and artificial intelligence, and further provides a future outlook for the development of reconfigurable electronics based on 2D material devices.

## 1. Introduction

Over the past decades, the trend of promoting the complementary metal-oxide-semiconductor (CMOS) technology integration level along with the predictions of Moore’s Law [1] has been facilitating the boost of computation power and the prosperity of artificial intelligence (AI). However, the rising demands of the data throughput and scaling down with the rapid development of big-data-processing and edge-computing scenarios are increasingly hard to be met, as conventional integrated circuit (IC) technology approaches its physical limit [2,3]. Therefore, extensive works have been conducted for innovative architectures, including devices based on silicon nanowires or nanosheets [4,5], carbon nanotubes [6,7], and two-dimensional (2D) semiconductor materials [8,9].

2D semiconductor materials have drawn increasing attention in recent years. 2D semiconductor materials are free of dangling bonds with ultra-thinned thickness and have less mobility degradation effects than silicon in the same thickness scale. 2D semiconductors with reduced thickness exhibit enhanced control of the channel current when the channel length is down to 10 nm, which promotes the transistor channel length scaling down [10,11]. The ultra-thinned bodies of 2D semiconductors enable easy electrostatic gating control of 2D semiconductor channels and channel polarity tuning between p-, n-, and intrinsic types [12]. 2D materials exhibit prominent physics properties, including confinement effects [13], optical responses [14], etc., which are promising for developing devices with extended functionalities. Large-area growth technologies of 2D semiconductor materials, including chemical vapor deposition [15], have been investigated to promote the applications of 2D semiconductors to large-scale integrated circuits. Recently, transistor structures in advanced nodes, including FinFETs [16] and GAAFETs [17], based on 2D semiconductor materials have been investigated to further enhance gate control, and three-dimensional (3D) integration designs of 2D material devices with higher area efficiency have been developed to validate the feasibility of 2D semiconductors for 3D stacking technology [18,19,20]. 2D materials are promising for the next-generation computational devices [21,22].

Recently, reconfigurable electronics based on 2D semiconductor materials have been investigated to overcome the physical limit and promote the area scaling down. 2D semiconductor channels with ambipolar characteristics can be easily doped as p-type or n-type channels by the gate voltage, and the channel current control dimension is increased at the device level [23,24,25]. 2D heterojunctions exhibit physical properties that modulate the transfer or output characteristics of channels and extend the functionalities of devices [26]. Reconfigurable electronics utilizing the properties of 2D semiconductors can change their functionalities to integrate multiple operations into circuit or device components, thereby reducing the number of transistors and increasing area efficiency [27,28]. Reconfigurable logic circuits based on 2D semiconductors have been developed to perform multiple types of logic operations, including basic logic like “AND” and “OR” gates and combinational logic like “multiplexer”, by highly simplified circuit forms, which largely reduces the required number of devices needed for reconfigurable logic operations [29,30,31]. 2D material devices with memory properties can switch their functions between transistor and memory, thereby integrating logic operation and data storage functions into the same device prototype [32]. Apart from digital logic operations, reconfigurable electronics based on 2D material devices have been investigated for analog computing of AI algorithms. Artificial synapses based on reconfigurable 2D material devices have been designed to emulate brain synaptic functions at the device level, which can be used for neuromorphic computing architectures [33]. 2D semiconductor channels with adjustable optical responses can be used to develop in-sensor computing architectures to reduce the data transmission pathway. Those architectures have been used for analog computing of artificial neural networks [34,35].

This work discusses reconfigurable electronics based on 2D semiconductor materials and their recent progress. Section 1 introduces the properties, preparation and growth technologies, and polarity control strategies of 2D semiconductor materials. Section 2 discusses 2D material devices with different mechanisms for reconfigurable functionalities. Section 3 and Section 4 analyze recently developed 2D-material-based reconfigurable electronics for logic operations and artificial intelligence, respectively. Finally, the future outlook of the development of reconfigurable electronics based on 2D material devices is given in Section 5.

## 2. Overview of 2D Semiconductor Materials

### 2.1. 2D Semiconductor Material Properties

**Figure 1:**
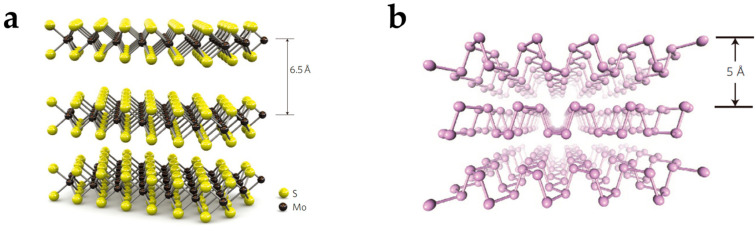
2D semiconductor structures. (**a**) The structure of MoS_2_ as a 2D TMDC material. The single-layer thickness is 6.5 Å. Reproduced with permission [36], copyright 2011, Springer Nature. (**b**) The structure of BP. The single-layer thickness is 5 Å. Reproduced with permission [37], copyright 2014, Springer Nature.

2D semiconductor materials have atomically thin bodies in each layer. Common 2D semiconductor materials include transition metal disulfide (TMDCs) (Figure 1a) [36] and black phosphorus (BP) (Figure 1b) [37]. Compared with bulk materials, 2D semiconductor materials have several prominent physical properties. With the reduction in channel material thickness for bulk materials like silicon, the degradation in mobility is severe due to the state of the surface trap and dangling bonds. On the contrary, the atomic arrangement of ultra-thinned 2D material layers has a pristine interface. The density of trap states is minimized, and scattering at channel–dielectric interfaces is reduced [38]. Therefore, 2D materials maintain good intrinsic mobility in atomical thickness. The reduction in channel thickness leads to the increase of the gate control capability in ultra-scale channel length, so 2D semiconductors with ultra-thinned thickness can be used to achieve short-channel transistors [39]. The thin body also enables easy electrostatic gating control to tune the energy band of the channel and change the polarity of materials [29]. 2D materials have good flexibility and mechanical strength to enable flexible electronics [40,41]. 2D semiconductors like MoS_2_ have ultra-high sensitivity in response to optical information [42], and 2D semiconductors can be used for high-performance photoelectric devices, including photodiodes [43] and spectrometers [44]. Two-dimensional semiconductor materials have drawn attention in the development of post-silicon transistor design and manufacturing [45].

### 2.2. Preparation and Growth Methods of 2D Semiconductor Materials

**Figure 2:**
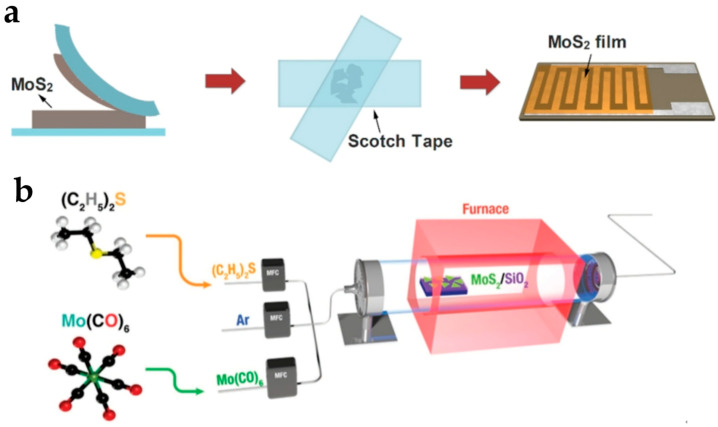
2D semiconductor fabrication methods. (**a**) Steps of mechanical exfoliation. Reproduced with permission [55], copyright 2019, MDPI AG. (**b**) Schematic diagram of MOCVD. Reproduced with permission [56], copyright 2021, Wiley-VCH.

Early discoveries and investigations on 2D materials are based on the mechanical exfoliation method [36,46]. Nowadays, mechanical exfoliation is commonly used to fabricate single or small-scale 2D material devices. The steps of mechanical exfoliation are shown in Figure 2a. First, the bulk crystal is pressed onto Scotch tape. Then, the Scotch tape is lifted to take 2D material flakes away from the bulk crystal, and few-layer 2D material flakes are located and transferred to the substrate by polydimethylsiloxane (PDMS). The transferred 2D material layer can be patterned by etching, and other fabrication processing like metal deposition can be conducted in the following steps. Mechanical exfoliation is a straightforward method to obtain thin-layer 2D materials. There are no chemical damage or high-temperature effects, and 2D materials made from mechanical exfoliation have excellent electrical and optical response properties and retain intrinsic physical properties [47].

Since there are problems of large device-to-device variability and hardness to achieve large-area synthesis, other methods have been developed for large-scale integration of 2D semiconductors. CVD is regarded as one of the most promising methods for the synthesis of large-area and single-layer two-dimensional materials [48]. CVD is an atomic-level surface modification process where a thin solid film is deposited on an underlying substrate through a chemical reaction from the vapor or gas phase. It usually has two steps, mass transport and a surface kinetics reaction [49]. CVD includes various branch methods, such as metal–organic CVD (MOCVD), plasma-enhanced CVD (PECVD), low-pressure CVD (LPCVD), etc. The schematic diagram of MOCVD is shown in Figure 2b. The high temperature used in CVD enables the high migration ability of atoms and molecules and promotes the growth of crystalline domains, which results in large domain sizes and good uniformity [50]. Main stream CVD growth methods have to use high temperatures to achieve good crystalline, while back-end-of-the-line technologies have maximum temperatures of about 500 °C [51]. A widely used solution is to transfer 2D material layer fabricated by CVD on a sapphire substrate to the chip. The transfer method of CVD 2D materials can be used for back-end-of-the-line technologies for wafer-scale production. Low-thermal-budget CVD synthesis approaches have also been developed in recent years [52]. Apart from mechanical exfoliation and CVD, other methods, including liquid phase exfoliation [53] and epitaxial growth [54], have been also used for the preparation and growth of 2D semiconductors.

### 2.3. Polarity of 2D Semiconductor Materials

**Figure 3:**
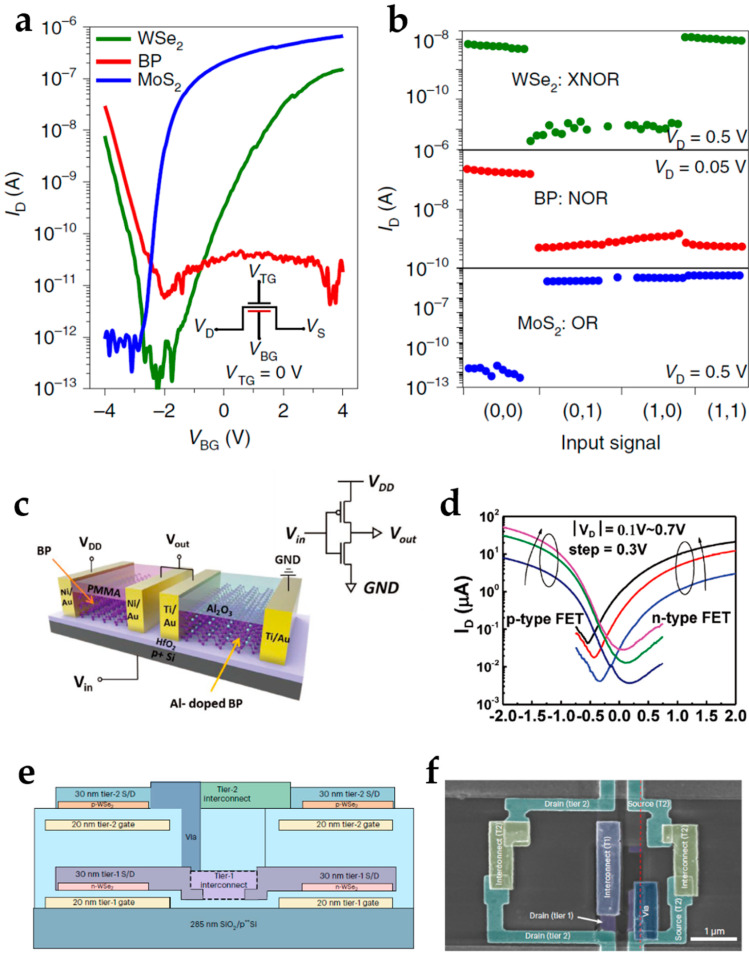
The polarity control of 2D semiconductor materials. (**a**) Different polarity properties of 2D semiconductor materials and (**b**) their corresponding operation applications. Reproduced with permission [57], copyright 2021, Springer Nature. (**c**,**d**) BP transistors with tunable polarities by the Al-donor doping technique of Al_2_O_3_ ALD processes. (**c**) The inverter with p-type BP and n-type Al-doped BP transistors. Reproduced with permission [62], copyright 2018, Wiley-VCH. (**d**) Transport curve of p-type and n-type BP transistors. Reproduced with permission [62], copyright 2018, Wiley-VCH. (**e**) The schematic and (**f**) scanning electron microscopy image of the complementary circuits composed of p-WSe2 and n-WSe2 by evaporations of contact metal with different work functions. Reproduced with permission [67], copyright 2024, Springer Nature.

Due to versatile physical properties of 2D semiconductors, different 2D semiconductor materials exhibit n-type, p-type, or ambipolar transport behaviors, thereby promoting the energy band engineering, and can achieve various operation functionalities. As shown in Figure 3a,b, the n-type MoS_2_ channel can be used for “OR” logic operation, the p-type BP channel can be used for “NOR” operation, and the ambipolar WSe_2_ channel can achieve “XNOR” logic operation [57]. Proper designs of device structures based on 2D semiconductors with different polarity properties can develop various reconfigurable electronics.

Polarities of 2D semiconductors, including WSe_2_ [58], MoTe_2_ [59], and BP [60], can be easily tuned since 2D semiconductors have ultra-thinned bodies. 2D semiconductor layers can exhibit ambipolar, unbalanced ambipolar, p-type, or n-type transport behaviors [61,62,63]. Various strategies can be applied to tune the mobilities and carrier densities of electrons and holes and change the semiconductors. For example, the atomic layer deposition (ALD) of high-k dielectric, including Al_2_O_3_, can enhance n-type behavior (Figure 3c,d).

To promote the applications of 2D materials in integrated circuits and very-large-scale integration, the performance of 2D semiconductor channels with both p- and n-type polarities should be enhanced to build complementary circuits. The development progress of high-performance n-type 2D channels like CVD-grown MoS_2_ is relatively advanced [64,65], but the development of pure p-type 2D channels is limited. There exits Fermi-level pinning near the conduction band caused by the interfacial defects induced by evaporation of the contact metal. Strategies to obtain high-performance p-type 2D channels have been developed in recent years by the stable evaporation of high-work-function metals [66]. Complementary circuits with n-type and p-type 2D material channels for 2D integrated circuits have been developed recently (Figure 3e,f) [67].

## 3. Working Principle of Reconfigurable Devices

Various strategies have been investigated for the implementation of reconfigurable devices to achieve different functionalities, including homojunction, heterojunction, and defects. Homojunction is formed by a 2D semiconductor material layer, which is more easily fabricated, and has fewer defects at the junction interface. The positions of junctions and the positions of gate boundaries are self-aligned. Heterojunctions are formed by the stacking of 2D materials. With proper structural designs, the modulation functions of heterojunctions can be tuned by a single gate. Heterojunctions deliver various physical properties due to the interactions of multiple 2D semiconductors. Defects in 2D material layers or 2D material device structures induce a charge trapping/de-trapping effect to implement programming or memory functionalities of reconfigurable electronics.

### 3.1. Homojunction

**Figure 4:**
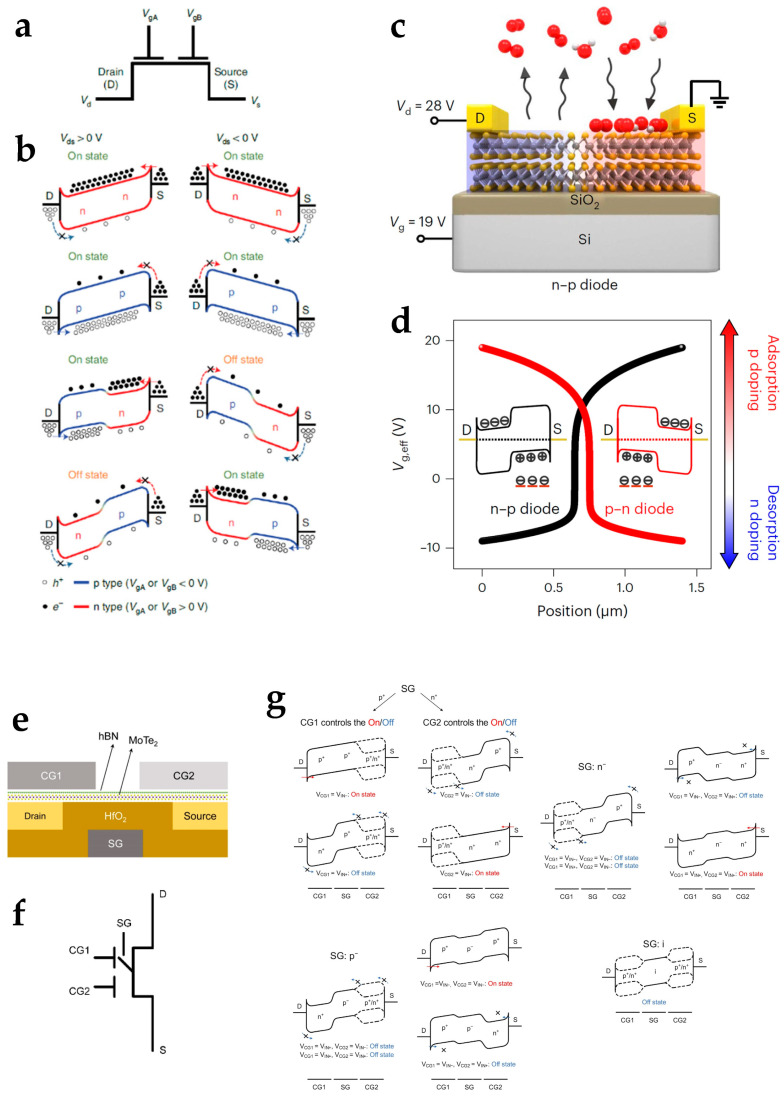
2D-material-based reconfigurable electronics based on homojunction structures. (**a**) The schematic symbol and (**b**) band diagrams of the electrically tunable two-dimensional homojunction for reconfigurable logic computing. Reproduced with permission [29], copyright 2020, Springer Nature. (**c**) The device schematic and (**d**) polarity switchable characteristics of the single-gate 2D material device implementing reconfigurable functions by graded doping. Reproduced with permission [74], copyright 2023, Springer Nature. (**e**) The device structure, (**f**) schematic symbol, and (**g**) band diagrams of the multibarrier collaborative modulation device. Reproduced with permission [31], copyright 2024, American Chemical Society.

A tunable homojunction can change the relations between the gate voltage and the drain current and therefore change the functionalities of devices. p–n homojunctions are widely used in 2D-material-based reconfigurable devices. A p–n homojunction with switchable polarities for ambipolar 2D materials can be used to build structures or circuits with various reconfigurable functions. A switchable p–n homojunction can be applied to device structures for circuits with reconfigurable logic computing (Figure 4a,b) [68,69]. Since photocurrents can be generated at p–n junctions, a switchable p–n homojunction can also change the direction of a photocurrent induced by light information and can be used for reconfigurable optoelectronic devices [70,71]. The doping of 2D materials can be conducted by ion implantation [72,73], but the polarity is unchanged after doping. Due to the ultra-thin body of 2D materials, electrostatic gating is an effective strategy to tune the energy band in a sufficiently large range and can dynamically change the polarity of the gate-controlled channel [12]. Other strategies for a switchable p–n homojunction have also been investigated. For example, Peng et al. proposed a single-gate reconfigurable device based on graded doping by the absorption and desorption of gas molecules (Figure 4c) [74]. The graded doping strategy can form a p–n homojunction for ambipolar MoTe_2_ with a single gate, which simplifies the device complexity (Figure 4d). Apart from a p–n homojunction, junctions with the same polarities, including n^+^–n^−^ or p^+^–p^−^ homojunctions, can also be used to achieve reconfigurable functions [75]. An n^+^–n^−^ or p^+^–p^−^ homojunction achieves various functionalities with less demand for the energy band tuning range. The integration of multiple forms of junctions can further enhance the functionality of reconfigurable structures. Pan et al. developed a multibarrier collaborative modulation architecture (Figure 4e,f) through interactions of p–n, n^+^–n^−^, and p^+^–p^−^ homojunctions (Figure 4g) and integrated combinational and reconfigurable logic computing functions at the device level [31].

### 3.2. Heterojunction

**Figure 5:**
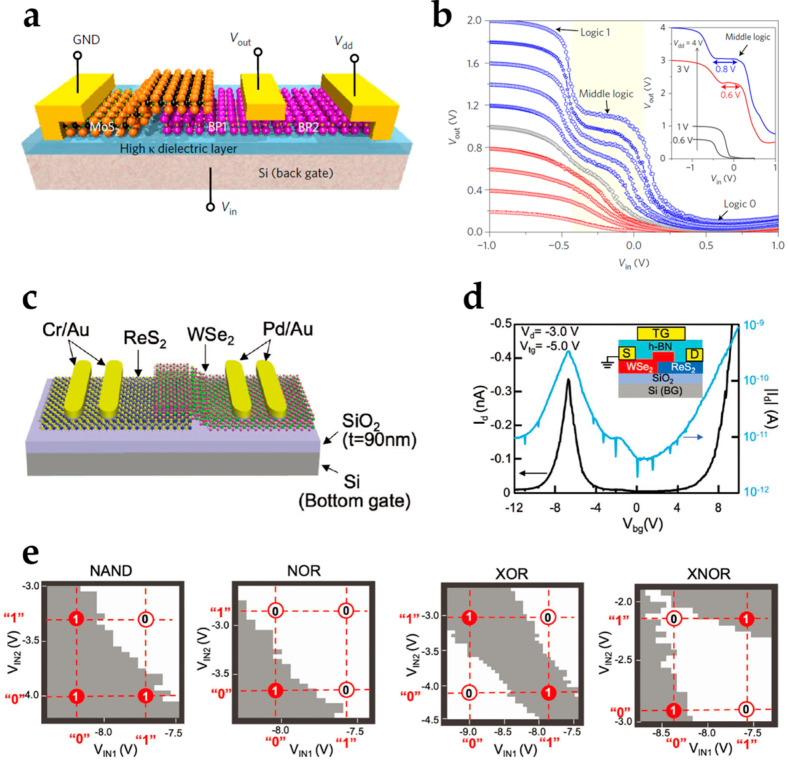
2D-material-based reconfigurable electronics based on heterojunctions. (**a**) A multifunctional BP/MoS_2_ heterojunction-based device for (**b**) tunable multi-value logic operations. Reproduced with permission [79], copyright 2017, Springer Nature. (**c**) A ReS_2_/WSe_2_ heterojunction-based reconfigurable device exhibiting (**d**) anti-ambipolar characteristics. Reproduced with permission [82], copyright 2022, Wiley-VCH. (**e**) Logic operations of the anti-ambipolar reconfigurable device to perform “NAND”, “NOR”, “XOR”, and “XNOR” logic. Reproduced with permission [82], copyright 2022, Wiley-VCH.

Heterojunctions have versatile electric modulation properties that can be applied to reconfigurable electronics. The unequal band gaps between two semiconductor materials induce more control dimensions for the transfer or output characteristics of devices. A commonly applied property is negative differential resistance (NDR). The energy bands of channels are variable by gate voltages, and the potential differences of the valence bands and conduction bands between two materials at the heterojunction are tunable [76]. When the tunable range is sufficiently large, the polarities of the potential differences can be altered, which can change the type of the major carrier in the channels or switch the relation between gate voltage and the carrier density [77,78]. Therefore, the curves of I–V characteristics exhibit different polarities of derivatives in different voltage intervals. 2D heterojunctions can be used for electronics with multi-valued logic operations. Huang et al. proposed a multifunctional device based on a BP/MoS_2_ heterojunction (Figure 5a) [79]. The NDR transfer characteristics enable the device to be used for a reconfigurable inverter that can process binary and ternary logic controlled by the supply voltage (Figure 5b). Types of logic operation functions can also be reconfigured by 2D heterojunctions. Seo et al. presented a BP/ReS_2_ heterojunction reconfigurable device [80]. NDR property is induced by the type-III BP/ReS_2_ heterojunction, and electron trapping/de-trapping is induced to tune the layer resistance. Reconfigurable circuits for ternary inverter and latch are built by the heterojunction. Another widely used property of a 2D heterojunction is negative differential transconductance or anti-ambipolar characteristics. The current is relative large for gate voltage that at the middle of the interval, and when increasing or decreasing the gate voltage, the current is reduced [81]. Shingaya et al. proposed a dual-gate anti-ambipolar reconfigurable device based on a ReS_2_/WSe_2_ heterojunction (Figure 5c,d) [82]. The anti-ambipolar device can achieve two-input logic operations of AND, OR, XOR, NAND, NOR, and XNOR (Figure 5e).

### 3.3. Charge Trapping/De-Trapping of Defects

**Figure 6:**
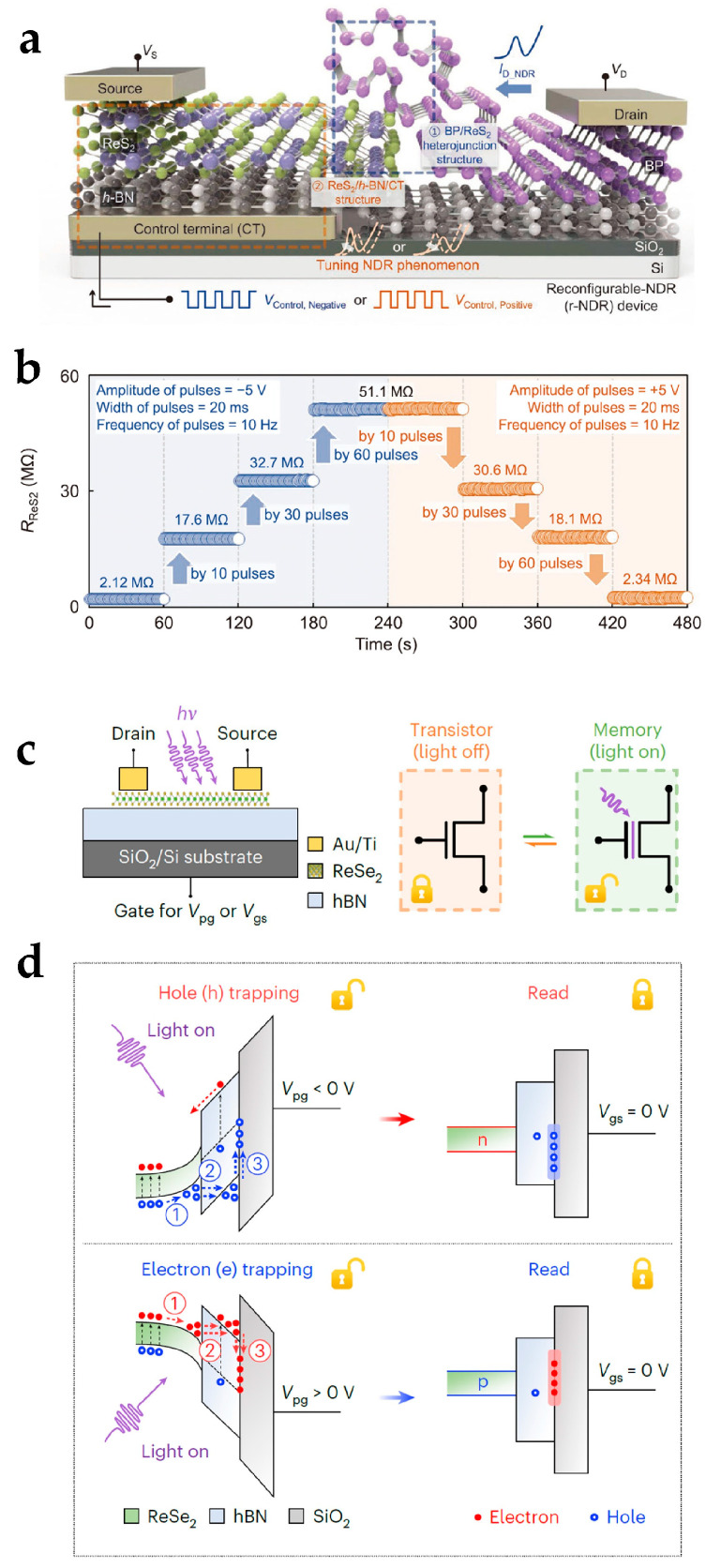
Defect functionalities in reconfigurable 2D material devices. (**a**) A reconfigurable device exhibiting tunable channel resistance by the charge trapping/de-trapping processes to or from vacancies in the ReS_2_ layer. Reproduced with permission [80], copyright 2022, Wiley-VCH. (**b**) The resistance change of the ReS_2_ layer by charge trapping/de-trapping under positive or negative electrical pulses. Reproduced with permission [80], copyright 2022, Wiley-VCH. (**c**) A reconfigurable transistor and memory with photoinduced trapping property at the dielectric interface. Reproduced with permission [32], copyright 2023, Springer Nature. (**d**) A schematic of the photoinduced trapping at the hBN/SiO_2_ interface. Reproduced with permission [32], copyright 2023, Springer Nature.

Defects in 2D material devices have been utilized in reconfigurable electronics. In 2D material layers, defects refer to structural abnormalities in a material due to the absence or irregular arrangement of atoms such as atomic vacancies or heteroatoms [83]. The defects of 2D TMDs are often expressed as intrinsic sulfur vacancies [84]. Defects of 2D material layers significantly affect the materials’ physical properties, including optical characteristics [85], electrical [86] and thermal [87] conductivity, etc. A widely applied design of utilizing the defects of 2D material layers is the control of charge trapping/de-trapping to or from the 2D material layer defects (Figure 6a) [80]. The 2D material layer conductivity is thereby adjusted (Figure 6b), and the I–V characteristics curves are tunable to achieve reconfigurable functionalities. Apart from defects in 2D material layers, charge trapping/de-trapping processes can be generated by interfaces of 2D material devices. Tsai et al. proposed a reconfigurable device based on a hBN/ReSe_2_/hBN heterostructure (Figure 6c) [32]. The non-volatile programmable functions of the device were based on the defect states in hBN and photoinduced trapping at the hBN/SiO_2_ interface. Under the control of the program gate voltage, holes or electrons tunneled through the hBN layer when the light signal was on and were trapped at the hBN/SiO_2_ interface (Figure 6d). The trapped charge exhibited non-volatile behavior when the light signal was off and electrostatically tuned the polarization states of the ReSe_2_ channel.

## 4. Two-Dimensional Semiconductor Reconfigurable Devices for Logic Operations

Recent research on logic devices based on two-dimensional semiconductor materials is of various functions and application scenarios. Reconfigurable logic devices have advantages, including area saving, low power consumption, etc. This section discusses the applications of 2D-material-based reconfigurable electronics for logic circuits.

### 4.1. Reconfigurable Logic Operation Circuits

**Figure 7:**
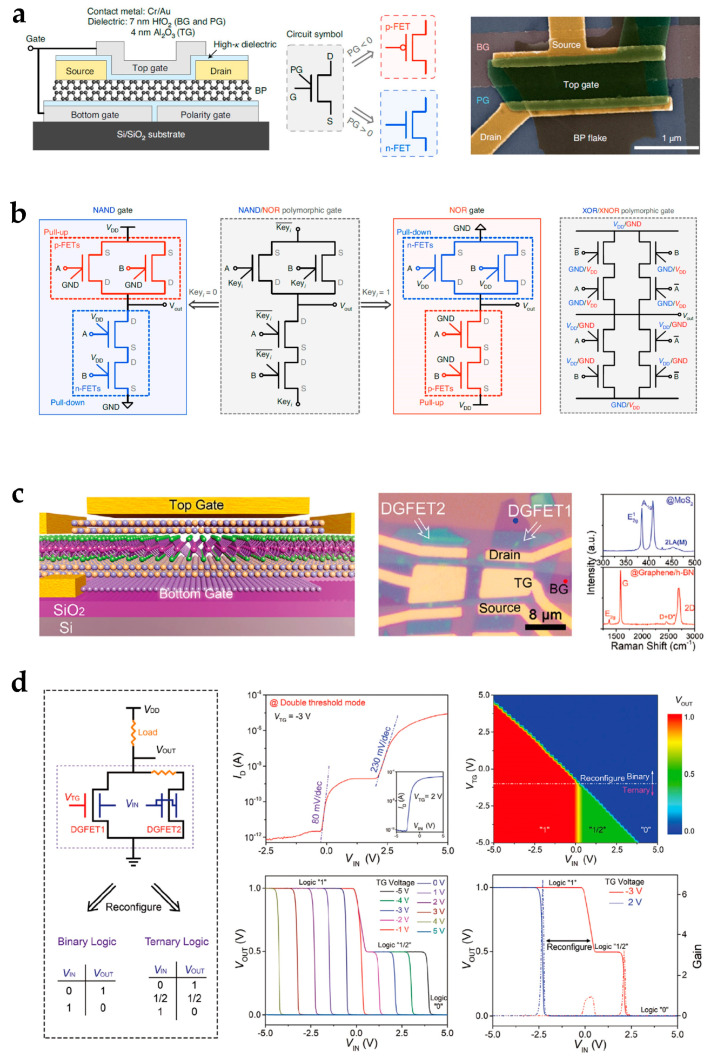
2D-material-based reconfigurable electronics for logic operation circuits. (**a**) Device characterizations and (**b**) NAND/NOR and XOR/XNOR polymorphic gates of the double-gate BP Schottky-barrier field effect transistor and circuits. Reproduced with permission [12], copyright 2020, Springer Nature. (**c**) Device characterizations and (**d**) logic functions of the double-gate transistor based on a MoS_2_ homojunction performing reconfigurable binary and ternary inverter circuit. Reproduced with permission [90], copyright 2021, Wiley-VCH.

Designs of reconfigurable electronics based on 2D material devices are investigated to promote the functionalities of reconfigurable logic circuits. Pan et al. proposed an electrically tunable homojunction device based on WSe_2_. The direction of the p–n junction and the drain current can be controlled by a combination of gate and drain voltage input polarity. When the polarities of the gate voltages were the same, the device channel appeared as n–n- or p–p-type doping. When the polarities were opposite, n–p or p–n junctions were formed, and multiple different current states were achieved. The device was used to build modules that have reconfigurable logic operation functions of two-input logic operations and multiplexer, d-latch, and adder/subtractor [29]. Wu et al. proposed a reconfigurable transistor based on a BP channel to build complementary logic gates (Figure 7a) [12]. This device exhibited programmable p-type and n-type transistor modes by adjusting the polarity gate voltage. Logic circuits were achieved to perform reconfigurable functions of “NAND/NOR” or “XOR/XNOR” operation functions (Figure 7b). Apart from increasing circuit functionalities, designs of 2D-material-based reconfigurable devices to increase area efficiency are also developed. Pan et al. reported a multibarrier collaborative modulation device for device-level high-density reconfigurable logic computing. Interactions of multiple forms of potential barriers were introduced to enhance the logic operation functionality at the device level. A percentage of 58.8% and 71.4% area is saved for combinational and reconfigurable logic operations [31]. To meet the demand for greater power saving and the reduction of system complexity in binary logic circuits, multi-valued logic circuits are attracting attention [88,89]. Yi et al. proposed a double-gate transistor architecture based on a MoS_2_ homojunction (Figure 7c) [90]. In the double-gate structure, the combination of top gate and bottom gate voltages controlled the charge-doping states of the channel. By adjusting the voltages, the charge-doping state had depletion, neutral, and accumulation modes. The device can be used as a circuit that can change the function between binary and ternary logic inverters by a gate voltage (Figure 7d).

### 4.2. Reconfigurable Logic and Memory Circuits

**Figure 8:**
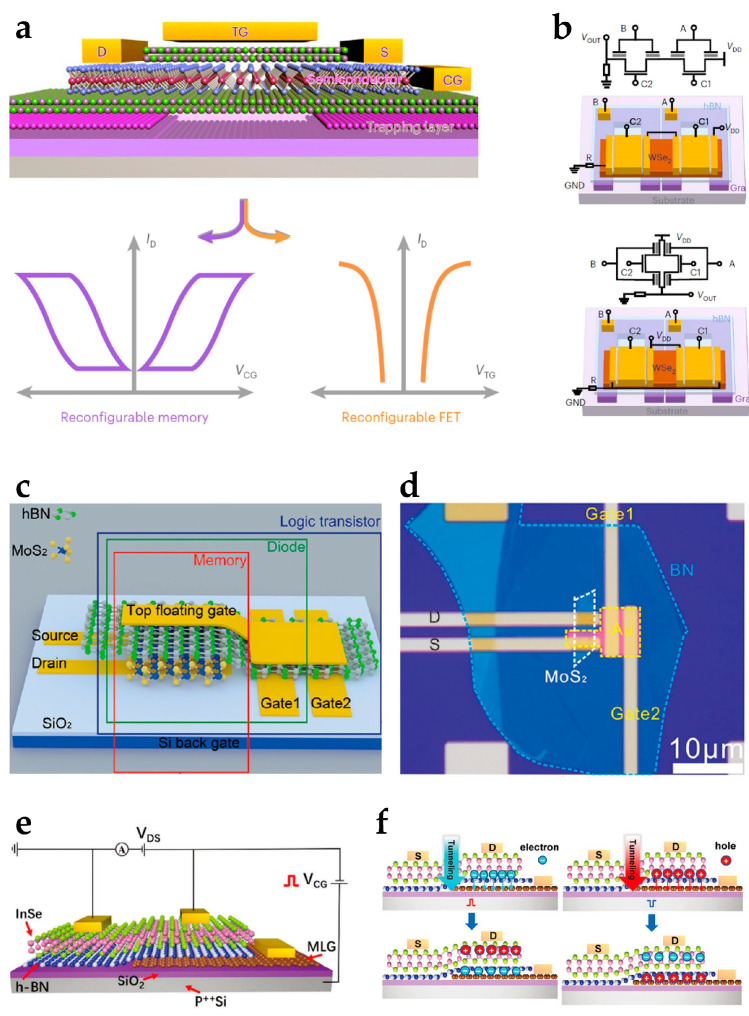
2D-material-based reconfigurable electronics for reconfigurable logic and memory circuits. (**a**) A partial-floating-gate architecture and (**b**) reconfigurable logic-in-memory circuits. Reproduced with permission [91], copyright 2022, Springer Nature. (**c**) The device schematic and (**d**) optical image of the side-gate BN-MoS_2_ transistor for reconfigurable operations of diode, reconfigurable logic, and memory. Reproduced with permission [92], copyright 2023, Wiley-VCH. (**e**) A semi-floating-gate transistor based on InSe homojunction with reconfigurable functions of rectifier, memory device, and ternary inverter. Reproduced with permission [93], copyright 2023, Wiley-VCH. (**f**) A schematic of electron and hole tunneling by voltage pulses. Reproduced with permission [93], copyright 2023, Wiley-VCH.

Strategies to integrate multiple-data-processing functionalities have been realized. Devices integrating logic operation and non-volatile memory were developed. Sun et al. developed a reconfigurable logic-in-memory architecture based on an ambipolar WSe_2_ homojunction and graphene partial floating gate (Figure 8a) [91]. The partial floating gate was used to store charge under specific voltage conditions. Homojunctions were formed between the channels controlled by the partial floating gate and the top gate, and the on/off states of the channel current were determined by the equality between the stored charge polarity and the top gate voltage. Reconfigurable circuits were developed to perform logic-in-memory operations of two-input logic (Figure 8b). Zeng et al. reported a side-gate reconfigurable device based on a MoS_2_ channel and hBN dielectric (Figure 8c,d) [92]. The conductivity of the channel was selectively regulated by side-gate voltages. Charge storage and erase at the top Au floating gate were implemented to achieve non-volatile memory. The device performed multiple functionalities, including diode, reconfigurable logic transistor, and floating-gate memory functions. Designs for the integration of multi-valued logic operations and memory functions were also proposed. Wang et al. proposed a semi-floating-gate-controlled 2D InSe homojunction (Figure 8e) [93]. A multilayer graphene floating gate performed charge storage. By applying a voltage pulse to the control gate of the silicon substrate, charge carriers tunneling through the hBN layer changed the carrier polarities of the charge stored in the multilayer graphene floating gate, forming different types of junctions in the InSe channel (Figure 8f). The structure implemented dynamic conversion between logic rectifiers, memories, and multi-valued logic inverters. Non-volatile memory functions have also been used for device programming to extend the logic operation functionalities of reconfigurable devices. Seo et al. proposed a ternary logic reconfigurable device latch based on a BP/ReS_2_ heterojunction [80]. Ternary logic operation is implemented by the NDR characteristics of the heterojunction, and electron trapping/de-trapping effects at ReS_2_ layer interfaces are utilized for logic function reconfiguration between ternary inverter and ternary latch operations.

### 4.3. Reconfigurable Optoelectronic Logic Circuits

**Figure 9:**
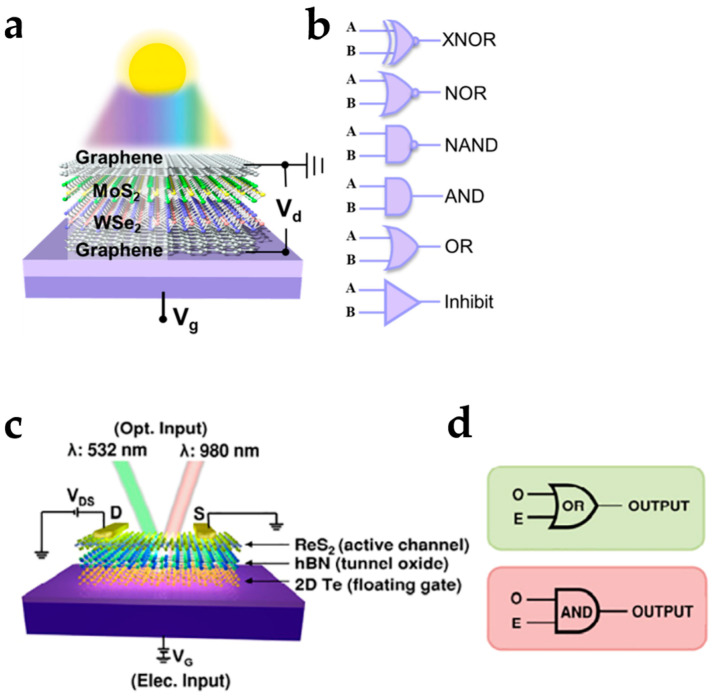
2D-material-based reconfigurable electronics for optoelectronic logic circuits. (**a**) A graphene/MoS_2_/WSe_2_/graphene heterojunction device for (**b**) optoelectronic reconfigurable logic gates. Reproduced with permission [94], copyright 2023, American Chemical Society. (**c**) A ReS2/hBN/2D Te floating-gate optoelectronic memory device for (**d**) optoelectronic logic operations of “OR” and “AND” logic. Reproduced with permission [95], copyright 2024, American Chemical Society.

Emerging optoelectronic technologies have made it possible for 2D semiconductor reconfigurable devices to be applied in the field of optoelectronic logic and memory devices. Ma et al. reported an optoelectronic reconfigurable logic device based on vertical field-effect transistors with a graphene/MoS_2_/WSe_2_/graphene heterojunction (Figure 9a) [94]. The device exhibited a reconfigurable modulation of photoresponse by gate and drain voltages. Optoelectronic reconfigurable logic gates of “XNOR”, “NOR”, “NAND”, “AND”, “OR”, and “Inhibit” were achieved (Figure 9b). Apart from defining optical responses as the outputs of logic gates, optical signals can also be the input of logic gates, where the optical signal and electrical signal are two of the logic gate inputs, and logic operations are implemented by combining the optical and electrical control to the device channel current. Bach et al. proposed a reconfigurable floating-gate optoelectronic memory that can be optically and electrically programmed by laser pulses and gate voltage pulses, respectively (Figure 9c) [95]. Based on the combined electrically and optically controllable charge storage properties, reconfigurable optical logic circuits, including “AND” and “OR” gates, were achieved, where laser and gate voltage pulses were two of the logic inputs, and the channel current was the output signal (Figure 9d). Optoelectronics logic operations are compatible with other functionalities, including optoelectronic artificial synaptic computing. A design to combine reconfigurable optoelectronics logic operations of “AND” and “OR” gates with optoelectronic artificial synapses was investigated to achieve a higher function integration of reconfigurable electronics [96].

## 5. 2D Semiconductor Reconfigurable Electronics for Artificial Intelligence

Reconfigurable electronics based on 2D materials have integrated functionalities and are compatible with the analog operations of AI algorithms. Therefore, extensive work has been conducted to develop reconfigurable electronics for AI operations to perform artificial synaptic computing or in-memory and in-sensor computing neural networks. For artificial synapse implementations, 2D material devices respond to the stimuli and output spike and pulse signals to emulate the electric signals of synapses. The output signals can be varied by the reconfiguration of the devices to emulate the variation of inhibitory and excitatory states of synapses. Artificial synapses can be integrated as an operation system to implement neuromorphic computing networks, including spiking neural networks. For in-memory and in-sensor computing neural network implementations, crossbar arrays composed of 2D material devices perform matrix-vector multiplications to implement analog computing. The states of the devices can be independently reconfigured in order to program the weight values of each cell in the network. This section analyzes the recent advances in 2D-material-based reconfigurable electronics for artificial synapses and in-memory/in-sensor computing neural networks.

### 5.1. 2D-Material-Based Reconfigurable Electronics for Artificial Synapses

**Figure 10:**
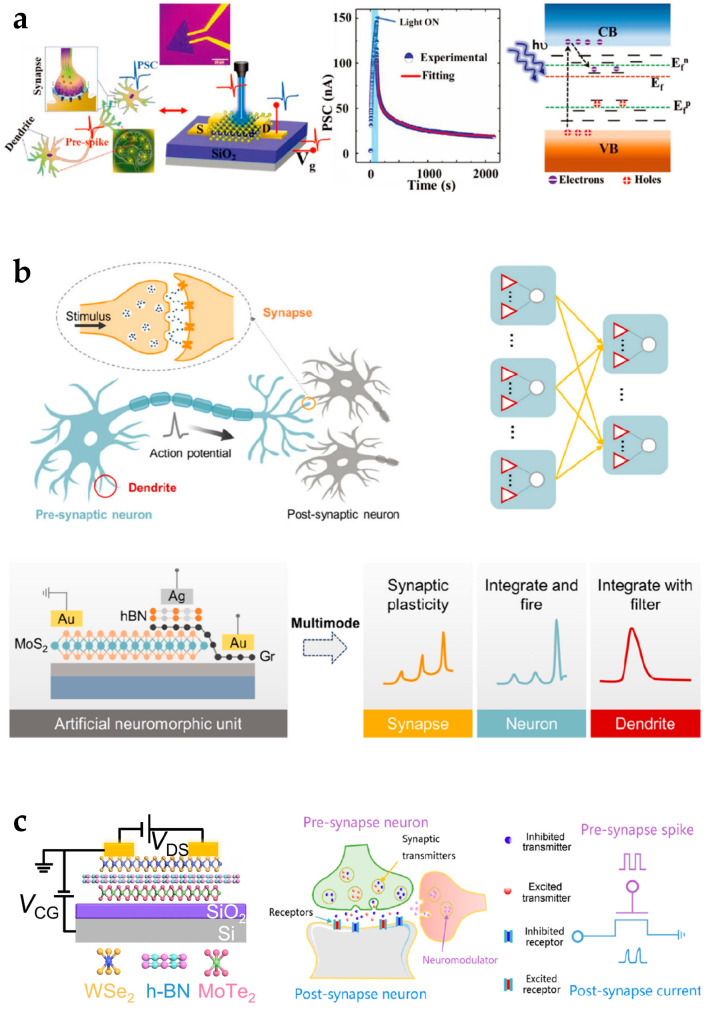
Artificial synapses based on 2D-material-based reconfigurable electronics. (**a**) 2D MoS_2_ artificial synapses for in-memory neuromorphic computing. Reproduced with permission [96], copyright 2022, Wiley-VCH. (**b**) Reconfigurable neuromorphic computing devices with multiple neural-information-processing functions. Reproduced with permission [99], copyright 2024, American Chemical Society. (**c**) A WSe_2_/hBN/MoTe_2_ artificial synapse emulating reconfigurable excitatory and inhibitory synaptic plasticity. Reproduced with permission [100], copyright 2023, American Chemical Society.

Artificial synapses aim to simulate the structure and function of biological neurons to process information in a parallel, pulse-driven manner, and 2D materials are promising for the hardware implementation of basic units of synapses [97,98]. Sahu et al. proposed a MoS_2_ optoelectronic artificial synapse based on a photoelectric cooperative stimulation mechanism (Figure 10a) [96]. The light pulse with different wavelengths was irradiated on the monolayer MoS_2_ material, which activated the photogenerated electron–hole pairs as charge carriers. Gate voltage was introduced to modulate the transport characteristics of charge carriers. Through photoelectric cooperative stimulation, this device imitated multiple biological neuromorphic behaviors, including long-term potentiation (LTP), and was used as neuromorphic computing architecture units. Hu et al. proposed a reconfigurable neuromorphic unit based on MoS_2_/Gr/hBN heterostructure, which emulated the function of key neural elements of the synapse, neuron, and dendrite (Figure 10b) [99]. The function of synapse and dendrite were stimulated by optical stimulation on a MoS_2_ photosensitive layer. The graphene electrode and the back gate electrode were used to receive excitatory and inhibitory input signals at the same time. The function of the neuron was stimulated by the Ag filament silver through the hBN layer to imitate the integrate-and-fire behavior of biological neurons, which showed the spike-based information coding ability. Yao et al. proposed a reconfigurable artificial synapse with a WSe_2_ channel and MoTe_2_ floating gate (Figure 10c) [100]. By adjusting the control voltage, the charge states in the floating gate were changed. The device exhibited properties of excitatory and inhibitory postsynaptic current, paired-pulse facilitation, and long-term potentiation and depression to emulate the functionalities of biological synapses.

### 5.2. 2D-Material-Based Reconfigurable Electronics for In-Memory and In-Sensor Computing Neural Networks

**Figure 11:**
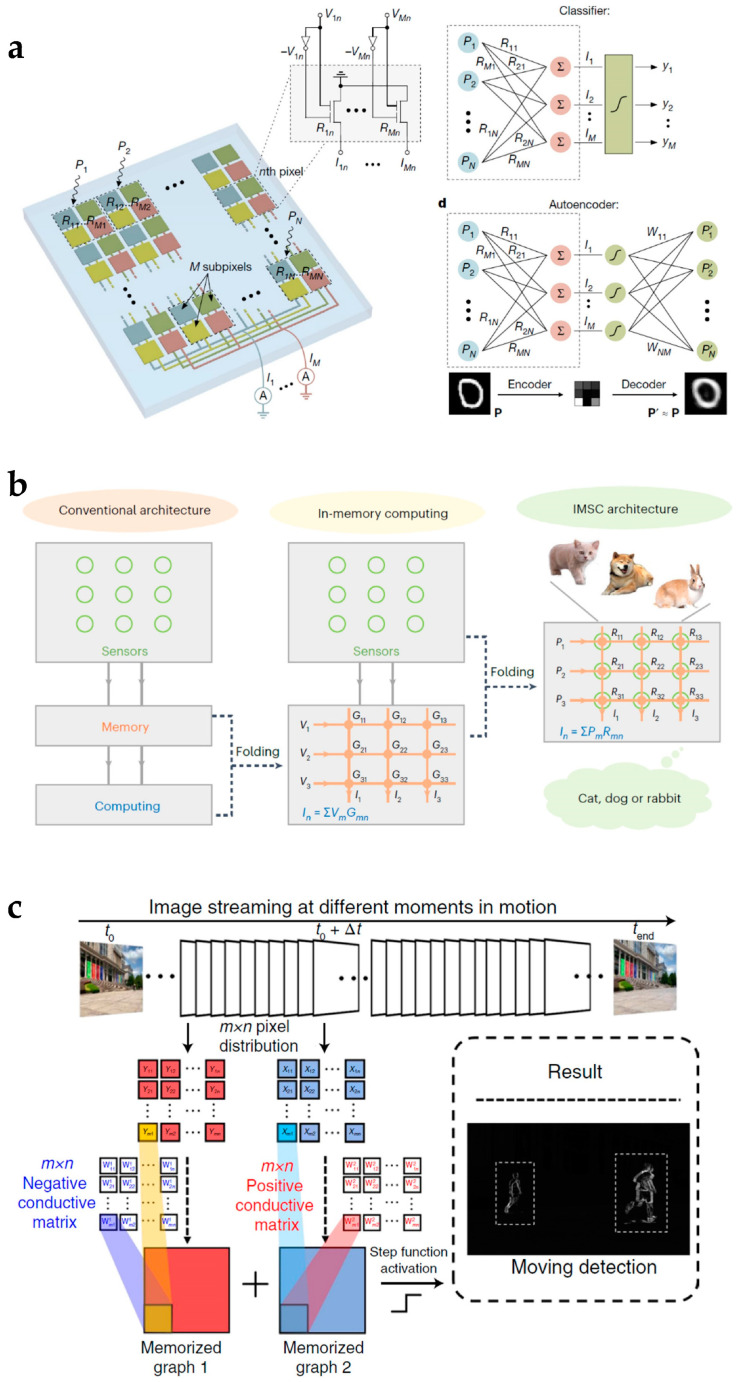
In-memory and in-sensor computing based on reconfigurable 2D material device. (**a**) Ultra-fast vision sensor based on WSe_2_ homojunction. Reproduced with permission [34], copyright 2020, Springer Nature. (**b**) In-memory sensing and computing neural network based on MoTe_2_ homojunction and (P(VDF-TrFE)) ferroelectric layer. Reproduced with permission [35], copyright 2023, Springer Nature. (**c**) All-in-one 2D retinomorphic device for motion recognition based on BP channel and WSe_2_ floating gate. Reproduced with permission [104], copyright 2022, Springer Nature.

The rapid development of artificial intelligence and edge computing brings growing demands for computational electronics. Conventional von Neumann architecture applied to most computational electronics is confronted with large time latency and power consumption due to the redundant pathway for information sensing, conversion, transmission, storage, and computing [101,102,103]. To address this problem, 2D material devices with reconfigurable functionalities have been investigated to enhance the performance of neural network processing by combining the functionalities of sensing, memory, and computing. Mennel et al. proposed an analog vision sensor based on a WSe_2_ homojunction (Figure 11a) [34]. The p–n homojunction direction was tunable with gate voltages, and the photocurrent direction can be programmed through gate voltage configuration. An analog computing architecture for image processing was established, and the directions of photocurrents were the weights of the neural network. The vision sensor implemented ultra-fast image processing with 40 ns. Wu et al. further extended the device-level functionalities of reconfigurable electronics and integrated sensing and memory functions into the device level through the MoTe_2_ layer with an organic ferroelectric poly(vinylidene fluoride) and trifluoroethylene (P(VDF-TrFE)) layer (Figure 11b) [35]. An in-memory sensing and computing architecture was developed. The applications of 2D-material-based reconfigurable electronics for in-memory and in-sensor computing have also been extended from static object recognition to motion detection. Zhang et al. proposed a 2D retinomorphic hardware based on a BP channel and WSe_2_ floating gate (Figure 11c) [104]. Based on the photoconductivity properties of WSe_2_, the device generated adjustable positive and negative responses to light stimuli and achieved moving target detection through inter-frame difference calculation.

## 6. Conclusions and Outlooks

Reconfigurable electronics can be applied to promote the function integration level and extend the data-processing functionalities for hardware performing logic operations and AI algorithms. 2D semiconductor materials can be used to develop high-performance reconfigurable electronics. This work reviews the basic properties of 2D materials, principles of 2D material devices for reconfigurable functionalities, and applications of 2D reconfigurable electronics. Presently, multiple structural designs of 2D-material-based reconfigurable electronics have been reported. The mainly utilized fundamental mechanisms of present reconfigurable electronics include homojunctions, heterojunctions, and the charge trapping/de-trapping of defects. In order to extend the functionalities of reconfigurable electronics, future research should exploit further physical properties of 2D semiconductors that have the potential to be used for function reconfiguration. One of the promising properties is the quantum effects of 2D semiconductors that may help to develop low-power or high-speed reconfigurable electronics. Reconfigurable 2D material devices have been investigated to be used for digital operations, including high-density reconfigurable logic circuits and hardware security modules and analog operations, including neuromorphic computing or in-sensor memory and computing. Research works have predicted the advanced performance of 2D-material-based reconfigurable electronics, including large area efficiency and low power consumption. Most presently proposed device or circuit prototypes were fabricated on relatively large sizes. In the future, fabrications of 2D-material-based reconfigurable electronics in advanced technology nodes should be developed to validate the performance advantages and the potential in practical applications. To achieve this objective, the stabilities of reconfigurable operations need to be further increased to meet the requirement of practical applications, and the variability of device units and different cycles should be reduced. Most present designs of reconfigurable 2D material devices are based on mechanical exfoliation. Large-area fabrication strategies of 2D-material-based reconfigurable electronics should be proposed to realize large-scale reconfigurable circuits.

## Data Availability

Not applicable.

## References

[1] Moore G.E. (1965). Cramming more components onto integrated circuits. Electronics.

[2] Cao W., Bu H., Vinet M., Cao M., Takagi S., Hwang S., Ghani T., Banerjee K. (2023). The future transistors. Nature.

[3] Liu A., Zhang X., Liu Z., Li Y., Peng X., Li X., Qin Y., Hu C., Qiu Y., Jiang H. (2024). The Roadmap of 2D Materials and Devices Toward Chips. Nano-Micro Lett..

[4] Mertens H., Ritzenthaler R., Hikavyy A., Kim M.S., Tao Z., Wostyn K., Chew S.A., De Keersgieter A., Mannaert G., Rosseel E. Gate-all-around MOSFETs based on vertically stacked horizontal Si nanowires in a replacement metal gate process on bulk Si substrates. Proceedings of the 2016 IEEE Symposium on VLSI Technology.

[5] Loubet N., Hook T., Montanini P., Yeung C.W., Kanakasabapathy S., Guillom M., Yamashita T., Zhang J., Miao X., Wang J. Stacked nanosheet gate-all-around transistor to enable scaling beyond FinFET. Proceedings of the 2017 Symposium on VLSI Technology.

[6] Franklin A.D., Hersam M.C., Wong H.P. (2022). Carbon nanotube transistors: Making electronics from molecules. Science.

[7] Zhang Z., Passlack M., Pitner G., Natani S., Su S.-K., Chao T.-A., Liew S.L., Hou V.D.H., Hsu C.-F., Shipley W.E. (2023). Complementary carbon nanotube metal–oxide–semiconductor field-effect transistors with localized solid-state extension doping. Nat. Electron..

[8] Wachter S., Polyushkin D.K., Bethge O., Mueller T. (2017). A microprocessor based on a two-dimensional semiconductor. Nat. Commun..

[9] Jayachandran D., Pendurthi R., Sadaf M.U.K., Sakib N.U., Pannone A., Chen C., Han Y., Trainor N., Kumari S., Mc Knight T.V. (2024). Three-dimensional integration of two-dimensional field-effect transistors. Nature.

[10] Shen Y., Dong Z., Sun Y., Guo H., Wu F., Li X., Tang J., Liu J., Wu X., Tian H. (2022). The Trend of 2D Transistors toward Integrated Circuits: Scaling Down and New Mechanisms. Adv. Mater..

[11] English C.D., Smithe K.K.H., Xu R.L., Pop E. Approaching ballistic transport in monolayer MoS_2_ transistors with self-aligned 10 nm top gates. Proceedings of the 2016 IEEE International Electron Devices Meeting (IEDM).

[12] Wu P., Reis D., Hu X.S., Appenzeller J. (2020). Two-dimensional transistors with reconfigurable polarities for secure circuits. Nat. Electron..

[13] Chen P., Atallah T.L., Lin Z., Wang P., Lee S.J., Xu J., Huang Z., Duan X., Ping Y., Huang Y. (2021). Approaching the intrinsic exciton physics limit in two-dimensional semiconductor diodes. Nature.

[14] Nassiri Nazif K., Daus A., Hong J., Lee N., Vaziri S., Kumar A., Nitta F., Chen M.E., Kananian S., Islam R. (2021). High-specific-power flexible transition metal dichalcogenide solar cells. Nat. Commun..

[15] Wu F., Tian H., Shen Y., Hou Z., Ren J., Gou G., Sun Y., Yang Y., Ren T.L. (2022). Vertical MoS_2_ transistors with sub-1-nm gate lengths. Nature.

[16] Chen M.L., Sun X., Liu H., Wang H., Zhu Q., Wang S., Du H., Dong B., Zhang J., Sun Y. (2020). A FinFET with one atomic layer channel. Nat. Commun..

[17] Chou B.J., Chung Y.Y., Yun W.S., Hsu C.F., Li M.Y., Su S.K., Liew S.L., Hou V.D., Chen C.W., Kei C.C. (2024). High-performance monolayer MoS_2_ nanosheet GAA transistor. Nanotechnology.

[18] Jiang J., Parto K., Cao W., Banerjee K. (2019). Ultimate Monolithic-3D Integration With 2D Materials: Rationale, Prospects, and Challenges. IEEE J. Electron Devices Soc..

[19] Guo Y., Li J., Zhan X., Wang C., Li M., Zhang B., Wang Z., Liu Y., Yang K., Wang H. (2024). Van der Waals polarity-engineered 3D integration of 2D complementary logic. Nature.

[20] Lu D., Chen Y., Lu Z., Ma L., Tao Q., Li Z., Kong L., Liu L., Yang X., Ding S. (2024). Monolithic three-dimensional tier-by-tier integration via van der Waals lamination. Nature.

[21] Asselberghs I., Smets Q., Schram T., Groven B., Verreck D., Afzalian A., Arutchelvan G., Gaur A., Cott D., Maurice T. Wafer-scale integration of double gated WS_2_-transistors in 300mm Si CMOS fab. Proceedings of the 2020 IEEE International Electron Devices Meeting (IEDM).

[22] Ahmed Z., Afzalian A., Schram T., Jang D., Verreck D., Smets Q., Schuddinck P., Chehab B., Sutar S., Arutchelvan G. Introducing 2D-FETs in Device Scaling Roadmap using DTCO. Proceedings of the 2020 IEEE International Electron Devices Meeting (IEDM).

[23] Tong L., Peng Z., Lin R., Li Z., Wang Y., Huang X., Xue K.H., Xu H., Liu F., Xia H. (2021). 2D materials-based homogeneous transistor-memory architecture for neuromorphic hardware. Science.

[24] Resta G.V., Balaji Y., Lin D., Radu I.P., Catthoor F., Gaillardon P.E., De Micheli G. (2018). Doping-Free Complementary Logic Gates Enabled by Two-Dimensional Polarity-Controllable Transistors. ACS Nano.

[25] Larentis S., Fallahazad B., Movva H.C.P., Kim K., Rai A., Taniguchi T., Watanabe K., Banerjee S.K., Tutuc E. (2017). Reconfigurable Complementary Monolayer MoTe_2_ Field-Effect Transistors for Integrated Circuits. ACS Nano.

[26] Meng Y., Wang W., Wang W., Li B., Zhang Y., Ho J. (2024). Anti-Ambipolar Heterojunctions: Materials, Devices, and Circuits. Adv. Mater..

[27] Fei W., Trommer J., Lemme M.C., Mikolajick T., Heinzig A. (2022). Emerging reconfigurable electronic devices based on two-dimensional materials: A review. InfoMat.

[28] Zhao Y., Sun H., Sheng Z., Zhang D.W., Zhou P., Zhang Z. (2023). Recent progress on ambipolar 2D semiconductors in emergent reconfigurable electronics and optoelectronics. Chin. Phys. B.

[29] Pan C., Wang C.-Y., Liang S.-J., Wang Y., Cao T., Wang P., Wang C., Wang S., Cheng B., Gao A. (2020). Reconfigurable logic and neuromorphic circuits based on electrically tunable two-dimensional homojunctions. Nat. Electron..

[30] Lee M., Park C.Y., Hwang D.K., Kim M.-g., Lee Y.T. (2022). Longitudinal and latitudinal split-gate field-effect transistors for NAND and NOR logic circuit applications. npj 2D Mater. Appl..

[31] Pan J., Wu F., Wang Z., Liu S., Guo P., Yin J., Zhao B., Tian H., Yang Y., Ren T.L. (2024). Multibarrier Collaborative Modulation Devices with Ultra-High Logic Operation Density. ACS Nano.

[32] Tsai M.-Y., Huang C.-T., Lin C.-Y., Lee M.-P., Yang F.-S., Li M., Chang Y.-M., Watanabe K., Taniguchi T., Ho C.-H. (2023). A reconfigurable transistor and memory based on a two-dimensional heterostructure and photoinduced trapping. Nat. Electron..

[33] Leong J.F., Fang Z., Sivan M., Pan J., Tang B., Zamburg E., Thean A.V.Y. (2023). N-P Reconfigurable Dual-Mode Memtransistors for Compact Bio-Inspired Feature Extractor with Inhibitory-Excitatory Spiking Capability. Adv. Funct. Mater..

[34] Mennel L., Symonowicz J., Wachter S., Polyushkin D.K., Molina-Mendoza A.J., Mueller T. (2020). Ultrafast machine vision with 2D material neural network image sensors. Nature.

[35] Wu G., Zhang X., Feng G., Wang J., Zhou K., Zeng J., Dong D., Zhu F., Yang C., Zhao X. (2023). Ferroelectric-defined reconfigurable homojunctions for in-memory sensing and computing. Nat. Mater..

[36] Radisavljevic B., Radenovic A., Brivio J., Giacometti V., Kis A. (2011). Single-layer MoS_2_ transistors. Nat. Nanotechnol..

[37] Li L., Yu Y., Ye G.J., Ge Q., Ou X., Wu H., Feng D., Chen X.H., Zhang Y. (2014). Black phosphorus field-effect transistors. Nat. Nanotechnol..

[38] Pal A., Chavan T., Jabbour J., Cao W., Banerjee K. (2024). Three-dimensional transistors with two-dimensional semiconductors for future CMOS scaling. Nat. Electron..

[39] Wu F., Ren J., Yang Y., Yan Z., Tian H., Gou G., Wang X., Zhang Z., Yang X., Wu X. (2021). A 10 nm Short Channel MoS_2_ Transistor without the Resolution Requirement of Photolithography. Adv. Electron. Mater..

[40] Li N., Wang Q., Shen C., Wei Z., Yu H., Zhao J., Lu X., Wang G., He C., Xie L. (2020). Large-scale flexible and transparent electronics based on monolayer molybdenum disulfide field-effect transistors. Nat. Electron..

[41] Tang J., Wang Q., Tian J., Li X., Li N., Peng Y., Li X., Zhao Y., He C., Wu S. (2023). Low power flexible monolayer MoS_2_ integrated circuits. Nat. Commun..

[42] Lopez-Sanchez O., Lembke D., Kayci M., Radenovic A., Kis A. (2013). Ultrasensitive photodetectors based on monolayer MoS_2_. Nat. Nanotechnol..

[43] Wu G., Wang X., Chen Y., Wu S., Wu B., Jiang Y., Shen H., Lin T., Liu Q., Wang X. (2020). MoTe_2_ p-n Homojunctions Defined by Ferroelectric Polarization. Adv. Mater..

[44] Wu G., Abid M., Zerara M., Cho J., Choi M., Coileáin C.Ó., Hung K.M., Chang C.R., Shvets I.V., Wu H.C. (2024). Miniaturized spectrometer with intrinsic long-term image memory. Nat. Commun..

[45] Zeng S., Liu C., Zhou P. (2024). Transistor engineering based on 2D materials in the post-silicon era. Nat. Rev. Electr. Eng..

[46] Novoselov K.S., Geim A.K., Morozov S.V., Jiang D., Zhang Y., Dubonos S.V., Grigorieva I.V., Firsov A.A. (2004). Electric field effect in atomically thin carbon films. Science.

[47] Li Y., Kuang G., Jiao Z., Yao L., Duan R. (2022). Recent progress on the mechanical exfoliation of 2D transition metal dichalcogenides. Mater. Res. Express.

[48] Lanza M., Smets Q., Huyghebaert C., Li L.J. (2020). Yield, variability, reliability, and stability of two-dimensional materials based solid-state electronic devices. Nat. Commun..

[49] Zhao T., Guo J., Li T., Wang Z., Peng M., Zhong F., Chen Y., Yu Y., Xu T., Xie R. (2023). Substrate engineering for wafer-scale two-dimensional material growth: Strategies, mechanisms, and perspectives. Chem. Soc. Rev..

[50] Sheng C., Dong X., Zhu Y., Wang X., Chen X., Xia Y., Xu Z., Zhou P., Wan J., Bao W. (2023). Two-Dimensional Semiconductors: From Device Processing to Circuit Integration. Adv. Funct. Mater..

[51] Briggs N., Subramanian S., Lin Z., Li X., Zhang X., Zhang K., Xiao K., Geohegan D., Wallace R., Chen L.-Q. (2019). A roadmap for electronic grade 2D materials. 2D Mater..

[52] Zhu J., Park J.H., Vitale S.A., Ge W., Jung G.S., Wang J., Mohamed M., Zhang T., Ashok M., Xue M. (2023). Low-thermal-budget synthesis of monolayer molybdenum disulfide for silicon back-end-of-line integration on a 200 mm platform. Nat. Nanotechnol..

[53] He Y., Andrade A.F., Menard-Moyon C., Bianco A. (2024). Biocompatible 2D Materials via Liquid Phase Exfoliation. Adv. Mater..

[54] Liu C., Liu T., Zhang Z., Sun Z., Zhang G., Wang E., Liu K. (2024). Understanding epitaxial growth of two-dimensional materials and their homostructures. Nat. Nanotechnol..

[55] Li W., Zhang Y., Long X., Cao J., Xin X., Guan X., Peng J., Zheng X. (2019). Gas Sensors Based on Mechanically Exfoliated MoS_2_ Nanosheets for Room-Temperature NO_2_ Detection. Sensors.

[56] Park J.H., Lu A.Y., Shen P.C., Shin B.G., Wang H., Mao N., Xu R., Jung S.J., Ham D., Kern K. (2021). Synthesis of High-Performance Monolayer Molybdenum Disulfide at Low Temperature. Small Methods.

[57] Chen H., Xue X., Liu C., Fang J., Wang Z., Wang J., Zhang D.W., Hu W., Zhou P. (2021). Logic gates based on neuristors made from two-dimensional materials. Nat. Electron..

[58] Das S., Appenzeller J. (2013). WSe_2_ field effect transistors with enhanced ambipolar characteristics. Appl. Phys. Lett..

[59] Lin Y.F., Xu Y., Wang S.T., Li S.L., Yamamoto M., Aparecido-Ferreira A., Li W., Sun H., Nakaharai S., Jian W.B. (2014). Ambipolar MoTe_2_ transistors and their applications in logic circuits. Adv. Mater..

[60] Zhu W., Yogeesh M.N., Yang S., Aldave S.H., Kim J.S., Sonde S., Tao L., Lu N., Akinwande D. (2015). Flexible black phosphorus ambipolar transistors, circuits and AM demodulator. Nano Lett..

[61] Du Y., Liu H., Deng Y., Ye P.D. (2014). Device perspective for black phosphorus field-effect transistors: Contact resistance, ambipolar behavior, and scaling. ACS Nano.

[62] Chen L., Li S., Feng X., Wang L., Huang X., Tee B.C.K., Ang K.W. (2018). Gigahertz Integrated Circuits Based on Complementary Black Phosphorus Transistors. Adv. Electron. Mater..

[63] Tian H., Li Y.-x., Li L., Wang X., Liang R., Yang Y., Ren T.-L. (2019). Negative Capacitance Black Phosphorus Transistors With Low SS. IEEE Trans. Electron Dev..

[64] Chen X., Xie Y., Sheng Y., Tang H., Wang Z., Wang Y., Wang Y., Liao F., Ma J., Guo X. (2021). Wafer-scale functional circuits based on two dimensional semiconductors with fabrication optimized by machine learning. Nat. Commun..

[65] Fan D., Li W., Qiu H., Xu Y., Gao S., Liu L., Li T., Huang F., Mao Y., Zhou W. (2023). Two-dimensional semiconductor integrated circuits operating at gigahertz frequencies. Nat. Electron..

[66] Wang Y., Kim J.C., Li Y., Ma K.Y., Hong S., Kim M., Shin H.S., Jeong H.Y., Chhowalla M. (2022). P-type electrical contacts for 2D transition-metal dichalcogenides. Nature.

[67] Pendurthi R., Sakib N.U., Sadaf M.U.K., Zhang Z., Sun Y., Chen C., Jayachandran D., Oberoi A., Ghosh S., Kumari S. (2024). Monolithic three-dimensional integration of complementary two-dimensional field-effect transistors. Nat. Nanotechnol..

[68] Wang F., Pei K., Li Y., Li H., Zhai T. (2021). 2D Homojunctions for Electronics and Optoelectronics. Adv. Mater..

[69] Li X., Zhou P., Hu X., Rivers E., Watanabe K., Taniguchi T., Akinwande D., Friedman J.S., Incorvia J.A.C. (2023). Cascaded Logic Gates Based on High-Performance Ambipolar Dual-Gate WSe_2_ Thin Film Transistors. ACS Nano.

[70] Zhou Y., Fu J., Chen Z., Zhuge F., Wang Y., Yan J., Ma S., Xu L., Yuan H., Chan M. (2023). Computational event-driven vision sensors for in-sensor spiking neural networks. Nature Electronics.

[71] Bu T., Duan X., Liu C., Su W., Hong X., Hong R., Zhou X., Liu Y., Fan Z., Zou X. (2023). Electrically Dynamic Configurable WSe_2_ Transistor and the Applications in Photodetector. Adv. Funct. Mater..

[72] Xu K., Zhao Y., Lin Z., Long Y., Wang Y., Chan M., Chai Y. (2017). Doping of two-dimensional MoS_2_ by high energy ion implantation. Semicond. Sci. Technol..

[73] Nipane A., Karmakar D., Kaushik N., Karande S., Lodha S. (2016). Few-Layer MoS_2_ p-Type Devices Enabled by Selective Doping Using Low Energy Phosphorus Implantation. ACS Nano.

[74] Peng R., Wu Y., Wang B., Shi R., Xu L., Pan T., Guo J., Zhao B., Song C., Fan Z. (2023). Programmable graded doping for reconfigurable molybdenum ditelluride devices. Nat. Electron..

[75] Jin T., Gao J., Wang Y., Zheng Y., Sun S., Liu L., Lin M., Chen W. (2022). Two-dimensional reconfigurable electronics enabled by asymmetric floating gate. Nano Res..

[76] Wu F., Tian H., Yan Z., Ren J., Hirtz T., Gou G., Shen Y., Yang Y., Ren T.L. (2021). Gate-Tunable Negative Differential Resistance Behaviors in a hBN-Encapsulated BP-MoS_2_ Heterojunction. ACS Appl. Mater. Interfaces.

[77] Shim J., Oh S., Kang D.H., Jo S.H., Ali M.H., Choi W.Y., Heo K., Jeon J., Lee S., Kim M. (2016). Phosphorene/rhenium disulfide heterojunction-based negative differential resistance device for multi-valued logic. Nat. Commun..

[78] Nourbakhsh A., Zubair A., Dresselhaus M.S., Palacios T. (2016). Transport Properties of a MoS_2_/WSe_2_ Heterojunction Transistor and Its Potential for Application. Nano Lett..

[79] Huang M., Li S., Zhang Z., Xiong X., Li X., Wu Y. (2017). Multifunctional high-performance van der Waals heterostructures. Nat. Nanotechnol..

[80] Seo S., Cho J.I., Jung K.S., Andreev M., Lee J.H., Ahn H., Jung S., Lee T., Kim B., Lee S. (2022). A Van Der Waals Reconfigurable Multi-Valued Logic Device and Circuit Based on Tunable Negative-Differential-Resistance Phenomenon. Adv. Mater..

[81] Li Y., Wang Y., Huang L., Wang X., Li X., Deng H.X., Wei Z., Li J. (2016). Anti-Ambipolar Field-Effect Transistors Based On Few-Layer 2D Transition Metal Dichalcogenides. ACS Appl. Mater. Interfaces.

[82] Shingaya Y., Zulkefli A., Iwasaki T., Hayakawa R., Nakaharai S., Watanabe K., Taniguchi T., Wakayama Y. (2022). Dual-Gate Anti-Ambipolar Transistor with Van der Waals ReS_2_/WSe_2_ Heterojunction for Reconfigurable Logic Operations. Adv. Electron. Mater..

[83] Cavallini M., Gentili D. (2022). Atomic Vacancies in Transition Metal Dichalcogenides: Properties, Fabrication, and Limits. Chempluschem.

[84] Zhou W., Zou X., Najmaei S., Liu Z., Shi Y., Kong J., Lou J., Ajayan P.M., Yakobson B.I., Idrobo J.C. (2013). Intrinsic structural defects in monolayer molybdenum disulfide. Nano Lett.

[85] Mahjouri-Samani M., Liang L., Oyedele A., Kim Y.S., Tian M., Cross N., Wang K., Lin M.W., Boulesbaa A., Rouleau C.M. (2016). Tailoring Vacancies Far Beyond Intrinsic Levels Changes the Carrier Type and Optical Response in Monolayer MoSe_2-x_ Crystals. Nano Lett.

[86] Wu X., Ge R., Akinwande D., Lee J.C. (2020). Understanding of multiple resistance states by current sweeping in MoS_2_-based non-volatile memory devices. Nanotechnology.

[87] Peng B., Ning Z., Zhang H., Shao H., Xu Y., Ni G., Zhu H. (2016). Beyond Perturbation: Role of Vacancy-Induced Localized Phonon States in Thermal Transport of Monolayer MoS_2_. J. Phys. Chem. C.

[88] Jo S.B., Kang J., Cho J.H. (2021). Recent Advances on Multivalued Logic Gates: A Materials Perspective. Adv. Sci. (Weinh.).

[89] Seo S., Koo J., Choi J.-W., Heo K., Andreev M., Lee J.-J., Lee J.-H., Cho J.-I., Kim H., Yoo G. (2021). Controllable potential barrier for multiple negative-differential-transconductance and its application to multi-valued logic computing. npj 2D Mater. Appl..

[90] Yi J., Sun X., Zhu C., Li S., Liu Y., Zhu X., You W., Liang D., Shuai Q., Wu Y. (2021). Double-Gate MoS_2_ Field-Effect Transistors with Full-Range Tunable Threshold Voltage for Multifunctional Logic Circuits. Adv. Mater..

[91] Sun X., Zhu C., Yi J., Xiang L., Ma C., Liu H., Zheng B., Liu Y., You W., Zhang W. (2022). Reconfigurable logic-in-memory architectures based on a two-dimensional van der Waals heterostructure device. Nat. Electron..

[92] Zeng D., Ding R., Liu G., Lu H., Zhang M., Xue Z., Tian Z., Di Z. (2023). Side-Gate BN-MoS_2_ Transistor for Reconfigurable Multifunctional Electronics. Adv. Electron. Mater..

[93] Wang H., Bao L., Guzman R., Wu K., Wang A., Liu L., Wu L., Chen J., Huan Q., Zhou W. (2023). Ultrafast-Programmable 2D Homojunctions Based on van der Waals Heterostructures on a Silicon Substrate. Adv. Mater..

[94] Ma Z., Yuan P., Li L., Tang X., Li X., Zhang S., Yu L., Jiang Y., Song X., Xia C. (2024). Optoelectronic Reconfigurable Logic Gates Based on Two-Dimensional Vertical Field-Effect Transistors. Nano Lett..

[95] Bach T.P.A., Cho S., Kim H., Nguyen D.A., Im H. (2024). 2D van der Waals Heterostructure with Tellurene Floating-Gate for Wide Range and Multi-Bit Optoelectronic Memory. ACS Nano.

[96] Sahu M.C., Sahoo S., Mallik S.K., Jena A.K., Sahoo S. (2022). Multifunctional 2D MoS_2_ Optoelectronic Artificial Synapse with Integrated Arithmetic and Reconfigurable Logic Operations for In-Memory Neuromorphic Computing Applications. Adv. Mater. Technol..

[97] Yoo C., Ko T.-J., Kaium M.G., Martinez R., Islam M.M., Li H., Kim J.H., Cao J., Acharya M., Roy T. (2022). A minireview on 2D materials-enabled optoelectronic artificial synaptic devices. APL Mater..

[98] Mukherjee S., Dutta D., Ghosh A., Koren E. (2023). Graphene-In_2_Se_3_ van der Waals Heterojunction Neuristor for Optical In-Memory Bimodal Operation. ACS Nano.

[99] Hu J., Li H., Zhang Y., Zhou J., Zhao Y., Xu Y., Yu B. (2024). Reconfigurable Neuromorphic Computing with 2D Material Heterostructures for Versatile Neural Information Processing. Nano Lett..

[100] Yao C., Wu G., Huang M., Wang W., Zhang C., Wu J., Liu H., Zheng B., Yi J., Zhu C. (2023). Reconfigurable Artificial Synapse Based on Ambipolar Floating Gate Memory. ACS Appl. Mater. Interfaces.

[101] Wang C.Y., Liang S.J., Wang S., Wang P., Li Z., Wang Z., Gao A., Pan C., Liu C., Liu J. (2020). Gate-tunable van der Waals heterostructure for reconfigurable neural network vision sensor. Sci. Adv..

[102] Zhang W., Gao B., Tang J., Yao P., Yu S., Chang M.-F., Yoo H.-J., Qian H., Wu H. (2020). Neuro-inspired computing chips. Nat. Electron..

[103] Dang Z., Guo F., Wang Z., Jie W., Jin K., Chai Y., Hao J. (2024). Object Motion Detection Enabled by Reconfigurable Neuromorphic Vision Sensor under Ferroelectric Modulation. ACS Nano.

[104] Zhang Z., Wang S., Liu C., Xie R., Hu W., Zhou P. (2022). All-in-one two-dimensional retinomorphic hardware device for motion detection and recognition. Nat. Nanotechnol..

